# Association of Cytokines in Individuals Sensitive and Insensitive to Dust Mites in a Brazilian Population

**DOI:** 10.1371/journal.pone.0107921

**Published:** 2014-09-19

**Authors:** Marcela Caleffi da Costa Lima Caniatti, Ariella Andrade Marchioro, Ana Lúcia Falavigna Guilherme, Luiza Tamie Tsuneto

**Affiliations:** 1 Post-Graduate Program in Health Sciences, Universidade Estadual de Maringá (UEM), Maringá, Paraná, Brazil; 2 Department of Basic Health Sciences, UEM, Maringá, Paraná, Brazil; Centro de Pesquisa Rene Rachou/Fundação Oswaldo Cruz (Fiocruz-Minas), Brazil

## Abstract

**Introduction:**

Allergic reaction to dust mites is a relatively common condition among children, triggering cutaneous and respiratory responses that have a great impact on the health of this population. Anaphylactic hypersensitivity is characterized by an exacerbated response involving the production of regulatory cytokines responsible for stimulating the production of IgE antibodies.

**Objective:**

To investigate an association of variants in cytokine genes (*IL1A* ^−889^, *IL1B* ^−511, +3962^, *IL1R* ^1970^, *IL1RA* ^11100^, *IL4RA* ^+1902^, *IL12* ^−1188^, *IFNG* ^+874^, *TGFB1* ^codon 10, codon 25^, *TNFA* ^−308, −238^, *IL2* ^−330, +166^, *IL4* ^−1098, −590, −33^, *IL6* ^−174, nt565^, and *IL10* ^−1082, −819, −592^) between patients sensitive to dust mites and a control group.

**Methods:**

A total of 254 patients were grouped as atopic and non-atopic according to sensitivity as evaluated by the Prick Test and to cytokine genotyping by the polymerase chain reaction-sequence specific primers (PCR-SSP) method using the Cytokine Genotyping Kit.

**Results:**

A comparison between individuals allergic to *Dermatophagoides farinae*, *Dermatophagoides pteronyssinus*, and *Blomia tropicalis* and a non-atopic control group showed significant differences between allele and genotype frequencies in the regulatory regions of cytokine genes, with important evidence for *IL4* ^−590^ in T/C (10.2% vs. 43.1%, odd ratio [OR] = 0.15, p = 5.2 10^−8^, pc = 0.0000011, and 95% confidence interval [95%CI] = 0.07–0.32) and T/T genotypes (42.9% vs. 13.8%, OR = 4.69, p = 2.5 10^−6^, pc = 0.000055, and 95%CI = 2.42–9.09). Other associations were observed in the pro-inflammatory cytokines *IL1A* ^−889^ (T/T, C, and T) and *IL2* ^−330^ (G/T and T/T) and the anti-inflammatory cytokines *IL4RA* ^+1902^ (A and G), *IL4* ^−590^ (T/C, T/T, C, and T), and *IL10* ^−592^ (A/A, C/A, A, and C).

**Conclusion:**

Our results suggest a possible association between single nucleotide polymorphisms (SNPs) in cytokine genes and hypersensitivity to dust mites.

## Introduction

The prevalence of allergic diseases has experienced an important increase in industrialized countries over recent decades. Although this increase has been widely investigated, the reasons for such trend have not yet been elucidated [1], [2]. According to the World Allergy Organization [3], children carry the greatest burden of the rising trend which has occurred over the past two decades. Despite this increase, in many countries there are no specialized services for patients with allergic diseases.

In respiratory allergies, such as asthma and rhinitis, there is inflammation of the airways as a result of an immune reaction, with release of cytoplasmic granules from active substances found in mast cells, basophils, and eosinophils [4], [5]. Two main factors contribute to the development and severity of allergic disease: host-related (genetic) factors and environmental factors, such as specific allergens, elements present in the environment, and air pollutants. When combined, these factors may trigger sensitization [6], [7].

In addition, there is the “hygiene hypothesis”, which became popular in the late 1980s to explain the high prevalence of atopic diseases in developed countries. It supports the idea that a reduced exposure to infections during childhood would predispose individuals to sensitization [8], [9], [10].

Since the mid-1960s, dust mites have been associated with allergic processes and identified as a causative agent of airway diseases, being considered one of the most important sources of allergens for humans. Among asthmatic patients, sensitivity to dust mites is present in 50% of adults and 80% of children [11], [12]. The term “house dust mite” has been adopted to describe mite species that can be found in the indoor environment and have the ability to elicit a response resulting in IgE antibodies [13].

The study of cytokines and sensitivity to dust mites is intended to corroborate the understanding of this relationship, given the high incidence of positive skin reactions to *Dermatophagoides pteronyssinus*, *D. farinae*, and *Blomia tropicalis* that has been commonly reported in the literature. It is noteworthy that these species belong to the class of arachnids (class Arachnida, subclass Acarina) [14], [15], [16] and have glycoproteins capable of inducing an anaphylactic response. However, little is known about the mechanism of action of these allergens against the development of immune response in certain individuals [17], [18].

Given the key role of cytokines in allergic reactions, gene variability in their regulatory regions might induce changes in the immune response [19]. Regulatory regions have shown an influence on cytokine production and transcription [20], [21], [22], [23]. Cytokines participate not only in the regulation of the immune response, but also directly in the inflammatory response [24]. There are pro-inflammatory cytokines (tumor necrosis factor alpha [TNF-α], interleukin [IL]-1 alpha [IL-1α], IL-1 beta [IL-1β], IL-2, IL-6, and interferon [IFN] gamma [IFN-γ]) and anti-inflammatory cytokines (IL-4, IL-10, tumor growth factor beta-1 [TGF-β1], and INF beta [IFN-β]) [25], [26], [27].

The involvement of interleukins in the pathogenesis of a range of diseases, such as lupus erythematosus, diabetes, chronic periodontitis, and cancer, has been widely studied. However, little is known about the association between single nucleotide polymorphisms (SNPs) in cytokine genes and sensitivity to dust mites. Therefore, we conducted a genetic association study to investigate markers of immune response in polymorphic variants of cytokine genes *IL1A* ^−889^ (rs1800587), *IL1B* ^−511, +3962^ (rs16944, rs1143634), *IL1R* ^1970^ (rs2234650), *IL1RA* ^11100^ (rs315952), *IL4RA* ^+1902^ (rs1801275), *IL12* ^−1188^ (rs3212227), *IFNG* ^+874^ (rs2430561), *TGFB1* ^codon 10, codon 25^ (rs1982073, rs1800471), *TNFA* ^−308, −238^ (rs1800629, rs361525), *IL2* ^−330, +166^ (rs2069762, rs2069763), *IL4* ^−1098, −590, −33^ (rs2243248, rs2243250, rs2070874), *IL6* ^−174, nt565^ (rs1800795, rs1800797), and *IL10* ^−1082, −819, −592^ (rs1800896, rs1800871, rs1800872) in mite-sensitive patients compared to mite-insensitive controls.

## Materials and Methods

### Ethical considerations

This study was approved by the Research Ethics Committee of Universidade Estadual de Maringá (protocol no. 693/2009) and conducted in accordance with the provisions of the Declaration of Helsinki. Educators and children from “Lar Escola de Maringá” and “Sistema de Apoio à Saúde São Rafael”, two local public institutions providing child care and health care respectively, were invited to participate in the study. All participants completed a registration form, were adequately informed about the project, and provided written informed consent prior to their inclusion in the study. In the case of minors, under 18 years of age, written consent was obtained from their parents/legal guardian.

### Sample size

Sample size calculation was based on city demographic data from the 2013 Census, with a population of 386,000 inhabitants. Assuming a 10% margin of error, maximum prevalence of 50% and 95% confidence interval, a minimum sample of 96 participants (cases) was required. Considering a possible loss of 10%, the final sample size was estimated at 138 participants (cases). Controls were defined according to the approximate number of cases, totaling 116 participants. Sample size was calculated using Statdisk version 8.4 [28].

### Inclusion and exclusion criteria

The study included all children and educators who voluntarily agreed to participate in the study.

### Characterization of the sample

Blood samples (10 ml) were drawn from 138 mite-sensitive and 116 mite-insensitive individuals, with a mean age of 12.9±7.6 years (range, 4–48 years), including 138 females and 116 males. Blood samples were collected into tubes containing EDTA anticoagulant.

### Allergy testing

A skin prick test (Prick Test) was used to check for immediate sensitization to antigens specific for dust mites. The participants were tested for the following allergens: *Dermatophagoides farinae*, *Dermatophagoides pteronyssinus*, and *Blomia tropicalis*. Dust mite and control extracts were applied at a distance of 2 cm from each other in a predetermined order. A lancet was used to gently prick the extracts onto the skin surface. Reactions were read after 15–20 min by two independent readers, and the size of the wheal or erythema was the classification criterion for the degree of allergic reaction. A reaction was recorded as positive if the diameter of the wheal or erythema was ≥5 mm and negative in the absence of these signs.

### Biological samples

Blood samples were centrifuged at 2500 rpm for 20 min to obtain the nucleated cell layer (buffy-coat), from which DNA was extracted using the commercial kit BIOPUR according to the manufacturer's instructions.

### Genotyping of polymorphic variants in cytokine genes

A NanoDrop (2000c/2000 UV-Vis) spectrophotometer was used to evaluate DNA purity and adjust its concentration. The polymerase chain reaction-sequence specific primer (PCR-SSP) technique was used to genotype allelic variants in cytokine genes, using a commercially available genotyping kit. The method consists of a sequence of specific primers capable of amplifying the product (or not) based on the presence (or absence) of SNP in each well. According to information provided by the manufacturer, in addition to determining genotypes, it is also possible to define the haplotypes of *TGFB1*, *TNFA*, *IL2*, *IL4*, *IL6*, and *IL10*. The 22 selected SNPs are listed in Table 1, including chromosomal locations and associated diseases as reported in previous studies.

**Table 1:** Cytokine genes associated with different diseases as reported in previous studies.

Gene	SNP	Chromosomal location	Diseases
*IL1A^−889^*	rs1800587	2q14	Irritant contact dermatitis [61]
*IL1B^−511^*	rs16944	2q14	Susceptibility to asthma [62]
*IL1B^+3962^*	rs1143634	2q14	Chronic periodontitis [63]
*IL1R^1970^*	rs2234650	2q14	Pulmonary tuberculosis [64]
*IL1RA* ^11100^	rs315952	2q14.2	Systemic lupus erythematosus[65]
*IL4RA* ^+1902^	rs1801275	16p12.1-p11.2	Eczema [66]
*IL12* ^−1188^	rs3212227	5q31.1-q33.1	Asthma [67]
*IFNG* ^+874^	rs2430561	12q14	Severe acute respiratory syndrome [68]
*TGFB1* ^codon 10^	rs1982073	19q13.1	Chronic obstructive pulmonary disease [69]
*TGFB1* ^codon 25^	rs1800471	19q13.1	Dust mite exposure and disease severity in children with asthma [70]
*TNFA* ^−308^	rs1800629	6p21.3	Chronic irritant contact dermatitis [50]
*TNFA* ^−238^	rs361525	6p21.3	Chronic rhinosinusitis[71]
*IL2* ^−330^	rs2069762	4q26-q27	Allergic disorders [53]
*IL2* ^+166^	rs2069763	4q26-q27	Lupus erythematosus; type 1 diabetes; cancer risk [72], [73], [74]
*IL4* ^−1098^	rs2243248	5q31.1	Allergic rhinitis and association with clinical phenotypes [75]
*IL4* ^−590^	rs2243250	5q31.1	Atopic asthma and allergic rhinitis; asthmatic children [57], [76]
*IL4* ^−33^	rs2070874	5q31.1	Atopic asthma and allergic rhinitis [57]
*IL6* ^−174^	rs1800795	7p21	Hypertension; type 2 diabetes; systemic-onset juvenile chronic arthritis [77], [78], [79]
*IL6* ^nt565^	rs1800797	7p21	Coronary artery disease [80]
*IL10* ^−1082^	rs1800896	1q31-q32	Dust mite exposure in allergy and asthma exacerbations; food allergy [43], [81]
*IL10* ^−819^	rs1800871	1q31-q32	Inflammatory and infectious respiratory diseases [82]
*IL10* ^−592^	rs1800872	1q31-q32	Cow's milk allergy [83]

SNP  =  single nucleotide polymorphism.

Each DNA sample, adjusted to a concentration of 20 to 200 ηg/µL, was added to the amplification solution provided in the kit, along with the Taq DNA polymerase enzyme, according to the volumes recommended by the manufacturer.

After the solution was homogenized, aliquots were poured onto polyethylene plates containing 48 wells with specific primers. Plates were then transferred to a thermal cycler for gene amplification, according to the manufacturer's instructions.

Fragments of the amplified DNA were separated by electrophoresis in a micro SSP gel System in a 3% gel solution and homogenized with 2 µL of SYBR safe stain at 90 volts for 15 min. DNA bands exposed to ultraviolet light were visualized by SYBR staining and the results were interpreted based on the presence or absence of the specific fragment of DNA. All 48 wells had an internal control of known size as informed by the manufacturer. The samples were photo-documented for reading of results.

### Statistical analysis

Allele, genotype and haplotype frequencies were determined by direct counting. Observed and expected frequencies of patients and controls were compared using a 2×2 contingency table. The significance of differences in allele, genotype and haplotype frequencies was evaluated using two-tailed Fisher's exact test with Yates' correction, followed by Bonferroni correction. The risk of developing sensitivity to dust mites was calculated by determining odds ratio (OR), according to Woolf [29], using Epi Info version 7. Hardy-Weinberg equilibrium (HWE) was tested using the method described by Guo and Thompson [30] with the Arlequin software version 3.0 (http://cmpg.unibe.ch/software/arlequin3) [31]. An age- and sex-adjusted logistic regression analysis was performed using SAS version 9.1 [32]. The level of significance for all analyses was set at p<0.05.

## Results

The frequency of cases (atopic individuals) and controls (non-atopic individuals) in males was 48.5% (67/138) and 42.2% (49/116) respectively. The logistic regression analysis showed no difference between males and females (p = 0.1579). As for age, the group of allergic individuals had a mean age of 13.9±8.5 years (range, 4–48 years), while controls had a mean age of 11.7±6.4 years (range, 5–37 years). According to the regression analysis, individuals aged 13–18 years were 2.75 times and those aged >18 years were 2.22 times more likely to be allergic (p = 0.0097 and p = 0.0358, respectively) than individuals under 12 years of age. The highest prevalence of allergy was observed in patients aged 13–18 years (Table 2).

**Table 2:** Results of age- and sex-adjusted logistic regression analysis.

	Participants					
Variables	n (cases)	n (total participants)	% cases	OR	95%CI	p
Age (years)						
≤12	86	178	48.3	Ref		
13–18	27	38	71.1	2.75	1.28–5.92	0.0097*
>18	25	38	65.8	2.22	1.06–4.65	0.0358*
Sex						
Male	67	116	57.8	Ref		
Female	71	138	51.4	0.69	0.41–1.15	0.1579

OR  =  odds ratio; Ref  =  reference; 95%CI  = 95% confidence interval;

* Significant at p<0.05.

Table 3 shows the results for allele, genotype and haplotype frequencies of *IL1A* ^−889^, *IL1B* ^−511, +3962^, *IL1R* ^1970^, *IL1RA* ^11100^, *IL4RA* ^+1902^, *IL12* ^−1188^, *IFNG* ^+874^, *TGFB1* ^codon 10, codon 25^, *TNFA* ^−308, −238^, *IL2* ^−330, +166^, *IL4* ^−1098, −590, −33^, *IL6* ^−174, nt565^, and *IL10* ^−1082, −819, −592^ in 138 patients sensitive to at least one of the dust mites (*D. farinae, D. pteronyssinus*, and *Blomia tropicalis*) and 116 controls.

**Table 3:** Significant allele, genotype and haplotype frequencies of cytokine SNPs in individuals allergic to at least one of the following dust mites: *Dermatophagoides farinae*, *Dermatophagoides pteronyssinus*, and *Blomia tropicalis*, and controls.

	Patients		Controls					
***IL1A*** **^−889^**	**n = 138**	**%**	**n = 116**	**%**	**p**	**pc**	**OR**	**95%CI**
C/C	80	58.0	56	48.3	ns	ns		
T/C	51	37.0	43	37.1	ns	ns		
T/T	7	5.1	17	14.7	0.0163	ns	0.31	0.12–0.78
C	211	76.4	155	66.8	0.0173	ns	1.61	1.09–2.38
T	65	23.5	77	33.2	0.0173	ns	0.62	0.42–0.91
***IL2^−^*** ^**330,+166**^	**n = 138**	**%**	**n = 116**	**%**	**p**	**pc**	**OR**	**95%CI**
GG/GG	8	5.8	10	8.6	ns	ns		
GG/TT	26	18.8	9	7.8	0.0109	ns	2.76	1.24–6.16
TG/GG	33	23.9	23	19.8	ns	ns		
TG/TG	29	21.0	32	34.4	ns	ns		
TG/TT	29	21.0	36	42.9	ns	ns		
TT/TT	13	9.4	6	7.1	ns	ns		
G/G^−330^	8	5.8	10	8.6	ns	ns		
G/T	59	42.7	32	27.6	0.0129	ns	1.96	1.15–3.33
T/T	71	51.4	74	63.8	ns	ns		
G	75	27.2	52	22.4	ns	ns		
T	201	72.8	180	77.6	ns	ns		
G/G^+166^	70	50.7	65	56.0	ns	ns		
G/T	55	39.9	45	38.8	ns	ns		
T/T	13	9.4	6	5.2	ns	ns		
G	195	70.6	175	75.4	ns	ns		
T	81	29.3	57	24.6	ns	ns		

SNPs  =  single nucleotide polymorphisms; p =  Fisher's exact test with Yates' correction; pc =  after Bonferroni correction; ns =  non-significant; OR =  odds ratio; 95%CI = 95% confidence interval.

A significant difference was observed in genotype and allele frequency between cases and controls at position −889 of the *IL1A* gene. The genotype T/T showed a negative association with sensitivity to dust mites (5.1% *vs.* 14.7%, OR = 0.31, p = 0.016, and 95% confidence interval [95% CI] = 0.12–0.78). An analysis of T allele variant revealed a negative association (23.5% *vs.* 33.2%, OR = 0.62, p = 0.017, and 95% CI = 0.42–0.91) with sensitivity to at least one of the three types of dust mites. The positions *IL2* ^−330, +166^ also showed significant frequency in the GG/TT haplotype and the G/T. The GG/TT haplotype and the G/T genotype were indicated as risk factors, with a frequency of 18.8% *vs.* 7.8% and 42.7% *vs.* 27.6% in the allergic group *vs.* the control group, respectively (Table 3).

When comparing the frequency of cytokine SNPs between 123 patients sensitive to dust mite 1 (*D. farinae*) and 116 controls, the *IL1A* ^−889^ SNP showed a statistically significant frequency in the T/T genotype (5.7% *vs.* 14.7%, OR = 0.35, p = 0.029, and 95% CI = 0.14–0.88) and in the T allele (23.6% *vs.* 33.2%, OR = 0.62, p = 0.025, and 95% CI = 0.41–0.93), with a negative association. The *IL4RA* gene at position +1902 also showed a significant frequency in the A and G alleles. While the A allele was indicated as a risk factor, the G allele showed a protective effect, with a frequency of 74.4% *vs.* 63.8% and 25.6% *vs.* 36.2% in the allergic group *vs.* the control group, respectively. In addition, *IL2* ^−330, +166^ SNPs also revealed significant haplotype and genotype frequencies, indicating the GG/TT haplotype and G/T genotype as risk factors, with a frequency of 18.7% *vs.* 7.8% and 43.1% *vs.* 27.6%, respectively. The genotype TT showed a significant frequency too, with 50.4% *vs.* 63.8% in atopic group *vs.* non-atopic group, respectively. Moreover, the *IL10* ^−592^ SNP showed a statistically significant difference in the frequency of A/A (34.1% *vs.* 13.8%, OR = 3.24, p = 0.00026, pc = 0.0058, and 95% CI = 1.70–6.18) and C/A genotypes (24.4% *vs.* 46.5%, OR = 0.37, p = 0.00041, pc = 0.0090, and 95% CI = 0.21–0.64) and in the frequency of A (46.3% *vs.* 37.1%, OR = 1.47, p = 0.0418, and 95% CI = 1.02–2.11) and C alleles (53.7% *vs.* 62.9%, OR = 0.68, p = 0.0418, and 95% CI = 0.47–0.98). These data suggest that individuals who express the A allele are at risk of developing hypersensitivity to dust mite 1, and those who express the C allele have a protective factor against this development (Table 4).

**Table 4:** Significant allele, genotype and haplotype frequencies of cytokine SNPs in individuals allergic to dust mite 1 (*Dermatophagoides farinae*) and controls.

	Patients		Controls					
***IL1A*** **^−889^**	**n = 123**	**%**	**n = 116**	**%**	**p**	**pc**	**OR**	**95%CI**
C/C	72	58.6	56	48.3	ns	ns		
T/C	44	35.8	43	37.1	ns	ns		
T/T	7	5.7	17	14.7	0.0299	ns	0.35	0.14–0.88
C	188	76.4	155	66.8	0.0251	ns	1.61	1.08–2.41
T	58	23.6	77	33.2	0.0251	ns	0.62	0.41–0.93
***IL4RA*** **^+1902^**	**n = 123**	**%**	**n = 116**	**%**	**p**	**pc**	**OR**	**95%CI**
A/A	70	56.9	52	44.8	ns	ns		
G/A	43	35.0	44	37.9	ns	ns		
G/G	10	8.1	20	17.2	ns	ns		
A	183	74.4	148	63.8	0.0133	ns	1.65	1.11–2.44
G	63	25.6	84	36.2	0.0133	ns	0.61	0.41–0.90
***IL2^−^*** ^**330, +166**^	**n = 123**	**%**	**n = 116**	**%**	**p**	**pc**	**OR**	**95%CI**
GG/GG	7	5.7	10	8.6	ns	ns		
GG/TT	23	18.7	9	7.8	0.0139	ns	2.73	1.21–6.19
GT/GT	1	0.8	0	0	ns	ns		
TG/GG	29	23.6	23	19.8	ns	ns		
TG/GT	1	0.8	0	0	ns	ns		
TG/TG	25	20.3	32	34.4	ns	ns		
TG/TT	27	21.9	36	42.9	ns	ns		
TT/TT	10	8.1	6	7.1	ns	ns		
G/G^−330^	8	6.5	10	8.6	ns	ns		
G/T	53	43.1	32	27.6	0.0149	ns	1.99	1.16–3.41
T/T	62	50.4	74	63.8	0.0497	ns	0.58	0.34–0.97
G	69	28.0	52	22.4	ns	ns		
T	177	71.9	180	77.6	ns	ns		
G/G^+166^	61	49.6	65	56.0	ns	ns		
G/T	51	41.5	45	38.8	ns	ns		
T/T	11	8.9	6	5.2	ns	ns		
G	173	70.3	175	75.4	ns	ns		
T	73	29.7	57	24.6	ns	ns		
***IL10^−^*** ^**1082, −819, −592**^	**n = 123**	**%**	**n = 116**	**%**	**p**	**pc**	**OR**	**95%CI**
ACA/ACA	1	0.8	1	0.9	ns	ns		
ACC/ACC	6	4.9	3	2.6	ns	ns		
ACC/ATA	24	19.5	28	24.1	ns	ns		
ATA/ATA	17	13.8	14	15.9	ns	ns		
GCC/ACC	36	29.3	38	37.2	ns	ns		
GCC/ATA	29	23.6	23	29.5	ns	ns		
GCC/GCC	8	6.5	5	6.4	ns	ns		
ACA/ATA	0	0	1	0.9	ns	ns		
ACC/ACA	1	0.8	1	0.9	ns	ns		
ATC/ATA	0	0	2	1.8	ns	ns		
ACC/ATC	1	0.8	0	0	ns	ns		
A/A^−1082^	50	40.6	50	43.1	ns	ns		
G/A	65	52.8	61	52.6	ns	ns		
G/G	8	6.5	5	4.3	ns	ns		
A	165	67.1	161	69.4	ns	ns		
G	81	32.9	71	30.6	ns	ns		
C/C^−819^	52	42.3	48	41.4	ns	ns		
C/T	54	43.9	52	44.8	ns	ns		
T/T	17	13.8	16	13.8	ns	ns		
C	158	64.2	148	63.8	ns	ns		
T	88	35.8	84	36.2	ns	ns		
A/A^−592^	42	34.1	16	13.8	0.000268	0.005896	3.24	1.70–6.18
C/A	30	24.4	54	46.5	0.00041	0.00902	0.37	0.21–0.64
C/C	51	41.5	46	39.7	ns	ns		
A	114	46.3	86	37.1	0.0418	ns	1.47	1.02–2.11
C	132	53.7	146	62.9	0.0418	ns	0.68	0.47–0.98

SNPs  =  single nucleotide polymorphisms; p =  Fisher's exact test with Yates' correction; pc =  after Bonferroni correction; ns =  non-significant; OR =  odds ratio; 95%CI = 95% confidence interval.

The analysis of the frequency of cytokine SNPs in 98 individuals sensitive to dust mite 2 (*D. pteronyssinus*) and 116 controls showed a significant difference in *IL2* ^−330, +166^ SNPs in the GG/TT haplotype (18.4% *vs.* 7.8%, OR = 2.67, p = 0.0234, and 95% CI = 1.14–6.26) and in the G/T genotype (42.9% *vs.* 27.6%, OR = 1.97, p = 0.0215, and 95% CI = 1.11–3.48). A positive association with sensitivity to dust mite 2 was found only at position −330. There was also a significant statistical difference in the *IL4* ^−590^ SNP when analyzing the distribution of genotypic and allelic variants. The T/C genotype (10.2% *vs.* 43.1%, OR = 0.15, p = 0.000000052, pc = 0.0000011, and 95% CI = 0.07–0.32) showed a negative association with sensitivity to dust mite 2, while the T/T genotype (42.9% *vs.* 13.8%, OR = 4.69, p = 0.0000025, pc = 0.000055, and 95% CI = 2.42–9.09) showed a positive association. Additionally, C and T alleles were indicated as protective and risk factors, respectively, for the development of sensitivity to dust mite 2, with a frequency of 52.0% *vs.* 64.7% and 48.0% *vs.* 35.3%, respectively (Table 5).

**Table 5:** Significant allele, genotype and haplotype frequencies of cytokine SNPs in individuals allergic to dust mite 2 (*Dermatophagoides pteronyssinus*) and controls.

	Patients		Controls					
***IL2^−^*** ^**330, +166**^	**n = 98**	**%**	**n = 116**	**%**	**p**	**pc**	**OR**	**95%CI**
GG/GG	6	6.1	10	8.6	ns	ns		
GG/TT	18	18.4	9	7.8	0.0234	ns	2.67	1.14–6.26
TG/GG	23	23.5	23	19.8	ns	ns		
TG/GT	1	1.0	0	0	ns	ns		
TG/TG	19	19.4	32	34.4	ns	ns		
TG/TT	21	21.4	36	42.9	ns	ns		
TT/TT	10	10.2	6	7.1	ns	ns		
G/G^−330^	6	6.1	10	8.6	ns	ns		
G/T	42	42.9	32	27.6	0.0215	ns	1.97	1.11–3.48
T/T	50	51.0	74	63.8	ns	ns		
G	54	27.5	52	22.4	ns	ns		
T	142	72.4	180	77.6	ns	ns		
G/G^+166^	48	49.0	65	56.0	ns	ns		
G/T	40	40.8	45	38.8	ns	ns		
T/T	10	10.2	6	5.2	ns	ns		
G	136	69.4	175	75.4	ns	ns		
T	60	30.6	57	24.6	ns	ns		
***IL4*** **^−1098, −590, −33^**	**n = 98**	**%**	**n = 116**	**%**	**p**	**pc**	**OR**	**95%CI**
GCC/GCC	1	1.0	2	1.7	ns	ns		
GTT/GTT	1	1.0	0	0	ns	ns		
TCC/GCC	11	11.2	10	8.6	ns	ns		
TCC/TCC	34	34.7	38	35.8	ns	ns		
TTC/TCC	5	5.1	7	9.0	ns	ns		
TTC/TTC	2	2.0	0	0	ns	ns		
TTT/GCC	3	3.1	11	14.1	ns	ns		
TTT/TCC	27	27.5	30	28.6	ns	ns		
TTT/TTC	4	4.1	3	3.5	ns	ns		
TTT/TTT	7	7.1	9	10.5	ns	ns		
GTC/GTC	0	0	1	0.9	ns	ns		
GTT/GCC	0	0	1	0.9	ns	ns		
GTT/GTC	0	0	1	0.9	ns	ns		
TTT/GTC	0	0	1	0.9	ns	ns		
TTT/GTT	1	1.0	1	0.9	ns	ns		
TTC/GCC	2	2.0	1	0.9	ns	ns		
G/G^−1098^	2	2.0	5	4.3	ns	ns		
T/G	17	17.3	24	20.7	ns	ns		
T/T	79	80.6	87	75.0	ns	ns		
G	21	10.7	34	14.7	ns	ns		
T	175	89.3	198	85.3	ns	ns		
C/C^−590^	46	46.9	50	43.1	ns	ns		
T/C	10	10.2	50	43.1	0.000000052	0.000001144	0.15	0.07–0.32
T/T	42	42.9	16	13.8	0.0000025	0.000055	4.69	2.42–9.09
C	102	52.0	150	64.7	0.0103	ns	0.59	0.40–0.87
T	94	48.0	82	35.3	0.0103	ns	1.68	1.14–2.49
C/C^−33^	55	56.1	59	50.9	ns	ns		
T/C	34	34.7	47	40.5	ns	ns		
T/T	9	9.2	10	8.6	ns	ns		
C	144	73.5	165	71.1	ns	ns		
T	52	26.5	67	28.9	ns	ns		

SNPs  =  single nucleotide polymorphisms; p =  Fisher's exact test with Yates' correction; pc =  after Bonferroni correction; ns =  non-significant; OR =  odds ratio; 95%CI = 95% confidence interval.

A total of 103 individuals were sensitive to dust mite 3 (*Blomia tropicalis*). The *IL2* ^−330, +166^ SNPs showed a significant frequency in the GG/TT haplotype (17.5% *vs.* 7.8%, OR = 2.52, p = 0.0387, and 95% CI = 1.08–5.89), being a risk factor for the development of sensitivity to dust mite 3 (Table 6).

**Table 6:** Significant allele, genotype and haplotype frequencies of cytokine SNPs in individuals allergic to dust mite 3 (*Blomia tropicalis*) and controls.

	Patients		Controls					
*IL2* ^−330,+166^	n = 103	%	n = 116	%	p	pc	OR	95%CI
GG/GG	7	6.8	10	8.6	ns	ns		
GG/TT	18	17.5	9	7.8	0.0387	ns	2.52	1.08–5.89
TG/GG	22	21.4	23	19.8	ns	ns		
TG/GT	1	1.0	0	0	ns	ns		
TG/TG	18	17.5	32	34.4	ns	ns		
TG/TT	25	24.3	36	42.9	ns	ns		
TT/TT	12	11.6	6	7.1	ns	ns		
G/G^−330^	7	6.8	10	8.6	ns	ns		
G/T	41	39.8	32	27.6	ns	ns		
T/T	55	53.4	74	63.8	ns	ns		
G	55	26.7	52	22.4	ns	ns		
T	151	73.3	180	77.6	ns	ns		
G/G^+166^	47	45.6	65	56.0	ns	ns		
G/T	44	42.7	45	38.8	ns	ns		
T/T	12	11.6	6	5.2	ns	ns		
G	138	67.0	175	75.4	ns	ns		
T	68	33.0	57	25.6	ns	ns		

SNPs  =  single nucleotide polymorphisms; p =  Fisher's exact test with Yates' correction; pc =  after Bonferroni correction; ns =  non-significant; OR =  odds ratio; 95%CI = 95% confidence interval.

As for the influence of regulatory regions on cytokine production, we found no significant difference in the association of *TGFB1*, *TNFA* and *IL10* genotypes and haplotypes between cases and controls as selected using the Cytokine Genotyping Kit (Invitrogen).

## Discussion

Allergy is a multifactorial condition, with the onset and severity dependent on genetic and environmental factors. Hypersensitivity to house dust mites may trigger different cutaneous and respiratory responses, which have a great impact on the health of affected individuals [33].

The discoveries made in the 1950s about the mechanisms of gene regulation in prokaryotes, such as lac or lambda repressor, have allowed researchers to investigate DNA binding sites in the regulatory regions of eukaryotes. Since then, several regulatory regions have been detected upstream and downstream of the transcribed gene, mainly in SNP regions.

The development of molecular biology techniques has allowed the identification of genetic polymorphisms within regulatory regions of cytokine genes, and also the finding that the level of cytokine production differs among individuals. These considerations have prompted many authors to investigate the regulation of genes expressing these cytokines in relation to susceptibility to and severity of different diseases [34], [35]. Although cytokines are usually related to allergic diseases, such as allergic rhinitis, allergic conjunctivitis, food allergy, atopic dermatitis, and asthma, they have been suggested as important biomarkers of various diseases, such as lupus erythematosus, pediatric ulcerative colitis [36], [37], [38], Alzheimer's disease [39], [40], [41], and ankylosing spondylitis [42].

There is an increasing number of studies devoted to this topic in the international literature. However, to date, there are no studies conducted in Brazil that address this issue. A cohort study of asthma in the Korean population suggested a possible involvement of *IL18* polymorphisms in asthma [33]. A study involving 417 Costa Rican children and 503 children participating in a childhood asthma management program showed significant changes in the interaction between IgE and dust mite allergens at three *IL10* SNP positions (rs1800896, rs3024492, and rs3024496), suggesting that exposure to these allergens significantly modified the effect of *IL10* SNPs [43]. Recently, higher serum levels of *TNFA* have been found in genotypes G/A and G/G of asthmatic children compared to controls (60.0% *vs.* 30%) [44].

There is considerable evidence associating interleukins and somatic complaints. Thompson and Barkhuizen [45] reported on alterations of *IL2*, *IL2* receptor (*IL2R*), *IL8*, and *IL1* receptor antagonist (*IL1RA*) in fibromyalgia and described the mechanism by which alterations in cytokines (in fibromyalgia and hepatitis C) may trigger hyperalgesia and other symptoms mediated by the presence of cytokine receptors on certain cells. They also pointed out the role of other cytokines, such as *IL6*, *IL10*, and *TNFA* in the relationship between chronic inflammation and mortality. A study carried out in Boston with 183 patients on hemodialysis showed a strong association between SNPs (*IL6* ^−174^, *IL10* ^−1082^, and *TNFA* ^−308^) and patients with end-stage renal disease [46].

Moreover, cytokines play an important physiological role in immune regulation and inflammatory processes, and changes in the aberrant expression or failure in production may contribute to the development of certain diseases [20], [21], [22], [23]. Because of the influence on cytokine production, these polymorphisms may provide flexibility to the immune response. In addition, the balance between pro- and anti-inflammatory cytokines is known to determine the intensity of inflammatory response [47].

The present results showed an important association between SNPs in cytokine genes and susceptibility to or protection from allergy to dust mites in *IL1A* at position −889, *IL4RA* at position +1902, *IL2* at positions −330 and +166, *IL4* at position −590, and *IL10* at position −592. All *loci* showed a gene frequency distribution in accordance with expected frequencies after calculating HWE, whose p-values were greater than the significance level of 0.05, *IL12* at position −1188 and *IL10* at position −1082. Considering that no evolutionary factor (mutation, migration, drift, or selection) was observed in this study, there may have been an error in the interpretation of the results. In order to avoid a type I error, the analyses of these two *loci* were excluded.

*IL1A* is synthesized by different cell types, such as activated macrophages and B lymphocytes, and by non-immune cells, such as fibroblasts. In addition, this cytokine is considered a potent inflammatory mediator and is located on chromosome 2q13 in humans [48]. Karjalainen *et al.* [49] conducted a study of 245 asthmatic adults and 405 controls associating the *IL1A* genotype with atopy in non asthmatic adults. No significant association was found between *IL1A* genotypes and skin test findings in asthmatic patients. Among controls, however, the *IL1A* genotype distribution was significantly different (p = 0.006) in individuals with positive (n = 150) and negative (n = 247) skin test responses. In our study, the T/T genotype and T allele of *IL1A* gene (rs1800587) were associated with protection against dust mite allergy (OR, 0.31 and 0.62 respectively) when cases were compared to controls. Conversely, the C allele was positively associated with disease (OR, 1.61).

In a German study [50], the *IL1A* −889 T allele was also associated with a protective effect, with a lower prevalence of dermatitis in a group exposed to a high risk of skin irritability. In that same study, the authors investigated the association between the *IL1A* −889 SNP and *IL1* cytokine levels in the stratum corneum. The *IL1* concentration was 23% and 47% lower in subjects with *IL1A* C/T and T/T genotypes, respectively, when compared to the C/C genotype. The reduced subcutaneous level of this pro-inflammatory cytokine for *IL1A* −889 T allele would explain the protective effect of this allele against dermatitis.

*IL2*, located on chromosome 4q27 in humans, is considered a key lymphokine, responsible for triggering the proliferation of Ly-T cells after interaction with peptide presentation [51], [52], which consequently stimulates Th2 responses and antibody production. Due to this central role in regulating the immune response, *IL2* is considered a strong candidate gene for allergic disease. Christensen *et al.* [53] investigated the involvement of *IL2* and *IL15* in the etiology of atopy and allergic diseases in a family-based association study of two Danish samples comprising a total of 235 families with at least two siblings with allergic disease. *IL2* at position −330 showed a significant association with three clinical forms of allergic disease (asthma, allergic rhinitis, and atopic dermatitis) in the group with 135 families, with p-values between 0.01 and 0.05, and *IL2* at position +166 also showed a significant association (with a p-value of 0.0005) in the same group. *IL15* showed no significant association.

SNP at position +166 does not alter *IL2* expression, because it does not interfere with the amino acid sequence, i.e., it is a silent mutation [54]. Therefore, the association occurs only at position −330.

Differences in cytokine production between allelic variants and their corresponding phenotypes were compared using Jonckheere's trend test for high, intermediate, and low comparisons. Individuals that were homozygous for the G allele produced over three times the amount of *IL2* than the T/T and T/G genotypes (p<0.001) [55]. In the present study, we observed significant differences in the frequency of the G/T and T/T genotypes, which are associated with low *IL2* production. The mechanism by which this is accomplished is currently under study. Given its influence on cell cycle advancement and apoptosis, much attention has been focused on the events that regulate *IL2* signal transduction. It is possible that the polymorphism at position −330 interferes with the binding of a key silencer element, thereby resulting in enhanced (or not) *IL2* production [55].

*IL4* and *IL4RA* are candidate genes for atopic diseases, i.e., diseases that are clinically expressed as atopic asthma, allergic rhinitis, and atopic dermatitis or that can be defined by at least one positive specific IgE response to inhalant or food allergens [56], [57]. A case-control study of 265 patients with persistent allergic rhinitis and 275 healthy controls revealed that the C/T and C/C genotypes in *IL4* ^−590^ were associated with a significantly decreased risk of mite-sensitized persistent allergic rhinitis (32.5% *vs.* 42.9%, OR = 0.64, 95%CI = 0.45-0.92) compared to the T/T genotype [56]. In the current study, a highly significant association was observed between the frequency of *IL4* ^−590^ SNP in 98 individuals allergic to *D. pteronyssinus* and 116 controls and the T/C genotype (p = 0.000000052) and the C allele (p = 0.010), suggesting a protective effect against sensitization to dust mites.

However, there are conflicting reports in the literature. Micheal *et al.* [57], in a case-control study of 214 atopic patients (108 with asthma and 106 with allergic rhinitis), found that the T/T genotype in the SNP rs2243250 was significantly associated with asthma (p  =  0.004) and allergic rhinitis (p <0.001), but playing a protective role rather than predisposing to respiratory allergy, as demonstrated by our data for the same genotype (p  =  0.0000025). Our results are consistent with findings of studies conducted in China [56], India [58], and Iran [59].

In Germany, Liu *et al.* [60] conducted a case-control study to examine the increased risk of sensitization to food, dust mites, cat epithelium, and outdoor allergens (e.g., pollen) in six SNPs (C-590T in the *IL4* gene, C-1055T and Arg130Gln in the *IL13* gene, and Ile50Val, Ser478Pro, and Gln551Arg in the *IL4RA* gene) and reported that, as for sensitization to dust mites, tests performed with 108 sensitized children and 432 controls revealed no significant association between sensitization to dust mite allergens and the six SNPs. Their findings disagree with our results for the *IL4RA* SNP (rs1801275), which expressed the A allele as a risk factor (OR, 1.65) and the G allele as a protective factor (OR, 0.61). It is worth noting that significant differences were observed between allele frequencies only for dust mite 1 (*D. farinae*), even after Bonferroni correction. No differences were observed in the genotypes at this position, probably due to the low risk obtained for these alleles.

The *IL10* ^−592^ gene was significantly different both for the A/A (OR, 3.24) and C/A (OR, 0.37) genotypes and for the A (OR, 1.47) and C (OR, 0.68) alleles. In Costa Rica, a study of 417 children and 503 participants of a childhood asthma management program highlighted the role of *IL10* in allergy and asthma exacerbations (when exposed to dust mites). Using a family-based approach to test for associations, the results showed that exposure to house dust mite allergens led to significant differences in the *IL10* gene [43].

So far, the present discussion has been based on the data considered significant as compared with those obtained from similar studies. However, because of the paucity of specific studies, we applied the Bonferroni correction to our data to avoid type II errors. After this correction, only *IL10* at position −592 for *D. farinae* and *IL4* at position −590 for *D. pteronyssinus* remained significant. These findings may suggest that different causative agents may be regulated by different cytokine production pathways, and that both cytokines have a biological role in controlling the production of different classes of antibodies. We believe that these data will add useful evidence to this line of research.

## Conclusions

Our results suggest an association of allelic and genotypic variants in the pro-inflammatory cytokines *IL1A* ^−889^ and *IL2* ^−330^ and anti-inflammatory cytokines *IL4* ^−590^, *IL4RA* ^+1902^ and *IL10* ^−592^.

The detection of candidate genes in individuals sensitive to dust mites may reveal mechanisms of interaction between allergies and hosts, and this detection allows the development of new preventive, diagnostic and therapeutic strategies. Thus, further studies are needed to clarify the role of these genetic markers in the susceptibility to and/or protection against house dust mite sensitivity.

## References

[1] Pastorino AC, Kuschnir FC, Arruda LKP, Casagrande RR, de Souza RG, et al (2008) Sensitisation to aeroallergens in Brazilian adolescents living at the periphery of large subtropical urban centres. Allergol Immunopathol 36: 9–16. 10.1157/13115665 18261427

[2] De Sario M, Galassi C, Biggeri A, Bisanti L, Ciccone G, et al (2005) Trends in the frequency of asthma and allergies. Epidemiol Prev 29: 86–90. 16128562

[3] World Allergy Organization (2011–2012) Who White Book on Allergy: Executive Summary. Available: http://www.worldallergy.org/publications/wao_white_book.pdf. Accessed 24 January 2013.

[4] Wallaert B (2011) Respiratory allergies. Soins 760: 38–41. 22216640

[5] Bush RK, Peden DB (2009) Advances in environmental and occupational disorders in 2008. J of Allergy and Clin Immunol 123: 575–578. 19281905 10.1016/j.jaci.2009.01.062

[6] Peden D, Reed CE (2012) Environmental and occupational allergies. J Allergy Clin Immunol 125: S150–S160. 10.1016/j.jaci.2009.10.073 20176257

[7] Sarinho ECS, Mariano J, Sarinho SW, Medeiros D, Rizzo JA, et al (2009) Sensitisation to aeroallergens among asthmatic and non-asthmatic adolescents living in a poor region in the Northeast of Brazil. Allergol Immunopathol 37: 239–243. 10.1016/j.aller.2009.03.009 19853356

[8] Kim DS, Drake-Lee AB (2003) Infection, allergy and the hygiene hypothesis: historical perspective. J Laryngol Otol 117: 946–950. 14738603 10.1258/002221503322683812

[9] Romagnani S (2004) The increased prevalence of allergy and the hygiene hypothesis: missing immune deviation, reduced immune suppression, or both? Immunology 112: 352–363. 15196202 10.1111/j.1365-2567.2004.01925.x PMC1782506

[10] Yazdanbakhsh M, Kremsner PG, Van Ree R (2002) Allergy, parasites, and the hygiene hypothesis. Science 296: 490–494. 11964470 10.1126/science.296.5567.490

[11] Atambay M, Aycan OM, Yologlu S, Karaman U, Daldal N (2006) The relationship between the skin allergy test and house dust mites. Turkiye Parazitol Derg 30: 327–329. 17309039

[12] Ertabaklar H, Yaman S, Ertug S (2006) Evaluation of the prevalence of house dust mites in house dust sent to the Adnan Menderes University Medical Faculty Parasitology Laboratory. Turkiye Parazitol Derg 30: 29–31. 17106851

[13] Fernandez-Caldas E (1997) Mite species of allergologie importance in Europe. Allergy 52: 383–387. 9188918 10.1111/j.1398-9995.1997.tb01016.x

[14] Morales-de-León G, López-García A, Arana-Muñoz O, Carcaño-Pérez Y, Papaqui-Tapia S, et al (2012) Correlación de reactividad cutánea entre extractos alergénicos de *Dermatophagoides pteronyssinus* y *Dermatophagoides farinae*, con *Blomia tropicalis* en pacientes con rinitis alérgica y asma. Rev Alerg Mex 59: 107–112. 24007986

[15] Medeiros Junior M, Figueiredo JP, Almeida MC, Atta AM, Taketomi EA, et al (2002) Association between Mite Allergen (Der p 1, Der f 1, Blo t 5) Levels and Microscopic Identification of Mites or Skin Prick Test Results in Asthmatic Subjects. Int Arch Allergy Immunol 129: 237–241. 12444321 10.1159/000066776

[16] Martínez Jiménez NE, Aguilar Angeles D, Rojas Ramos E (2010) Sensitization to Blomia tropicalis and Dermatophagoides pteronyssinus, farinae and siboney prevalence in patients with rhinitis, allergic asthma, or both, in a population of a metropolitan área of Mexico City. Rev Alerg Mex 57: 3–10. 20857623

[17] Petersen A, Schramm G, Schlaak M (1998) Post-translational modifications influence IgE reactivity to the allergen PhI p1 of Timothy grass pollen. Clin Exp Allergy 28: 315–321. 9543081 10.1046/j.1365-2222.1998.00221.x

[18] Weber A, Schröder H, Thalberg K, März L (1987) Specific interaction of IgE antibodies with a carbohydrate epitope of honey bee venom phospholipase A2. Allergy 42: 464–470. 2444130 10.1111/j.1398-9995.1987.tb00364.x

[19] Read RC, Camp NJ, di Giovine FS, Borrow R, Kaczmarski EB, et al (2000) An interleukin-1 genotype is associated with fatal outcome of meningococcal disease. J Infect Dis 182: 1557–1560. 11023482 10.1086/315889

[20] Meenagh A, Williams F, Ross OA, Patterson C, Gorodezky C, et al (2002) Frequency of cytokine polymorphisms in populations from Western Europe, Africa, Asia, the Middle East and South America. Hum Immunol 63: 1055–1061. 12392859 10.1016/s0198-8859(02)00440-8

[21] Pravica V, Asderakis A, Perrey C, Hajeer A, Sinnott PJ, et al (1999) In vitro production of IFN-gamma correlates with CA repeat polymorphism in the human IFN-gamma gene. Eur J Immunogenet 26: 1–3. 10068907 10.1046/j.1365-2370.1999.00122.x

[22] Fishman D, Faulds G, Jeffery R, Mohamed-Ali V, Yudkin JS, et al (1998) The effect of novel polymorphisms in the interleukin-6 (IL-6) gene on IL-6 transcription and plasma IL-6 levels, and an association with systemic-onset juvenile chronic arthritis. J Clin Invest 102: 1369–1376. 9769329 10.1172/JCI2629 PMC508984

[23] Turner DM, Williams DM, Sankaran D, Lazarus M, Sinnott PJ, et al (1997) An investigation of polymorphism in the interleukin-10 gene promoter. Eur J Immunogenet 24: 1–8. 10.1111/j.1365-2370.1997.tb00001.x 9043871

[24] Van Der Meide PH, Schellekens H (1996) Cytokines and the immune response. Biotherapy 8: 243–249. 8813336 10.1007/BF01877210

[25] Cianciarullo MA, Ceccon MEJ, Yamamoto L, Del Negro GMB, Okay TS (2008) Pro-inflammatory and anti-inflammatory mediators in neonatal sepsis: association between homeostasis and clinical outcome. J Hum Growth Develop 18: 135–147.

[26] Bélanger M, Allaman I, Magistretti PJ (2011) Differential effects of pro- and anti-inflammatory cytokines alone or in combinations on the metabolic profile of astrocytes. J Neurochem 116: 564–576. 21143598 10.1111/j.1471-4159.2010.07135.x

[27] Cerwenka A, Lanier LL (2001) Natural killer cells, viruses and cancer. Nat Rev Immunol 1: 41–49. 11905813 10.1038/35095564

[28] Triola MF (2003) Statdisk (version 8.4) for Elementary Statistics. Rio de Janeiro: LTC. 410p.

[29] Woolf B (1995) On estimating the relation between blood group and disease. Ann Hum Genet 19: 251–253. 10.1111/j.1469-1809.1955.tb01348.x 14388528

[30] Guo SW, Thompson EA (1992) A Monte Carlo method for combined segregation and linkage analysis. Am J Hum Genet 51: 1111–1126. 1415253 PMC1682838

[31] Excoffier L, Laval G, Schneider S (2005) Arlequin (version 3.0): An integrated software package for population genetics data analysis. Evol Bioinform Online 1: 47–50. PMC2658868 19325852

[32] Barr A, Goodnight J, Sall J, Helwig J (1999) SAS Software (version 9.1). North Carolina: SAS Institute Inc.

[33] Shin HD, Kim LH, Park BL, Choi YH, Park HS, et al (2005) Association of interleukin 18 (IL18) polymorphisms with specific IgE levels to mite allergens among asthmatic patients. Allergy 60: 900–906. 15932380 10.1111/j.1398-9995.2005.00619.x

[34] Hollegaard MV, Bidwell JL (2006) Cytokine gene polymorphism in human disease: on-line databases, Supplement 3. Genes Immun 7: 269–276. 16642032 10.1038/sj.gene.6364301

[35] Bidwell J, Keen L, Gallagher G, Kimberly R, Huizinga T, et al (1999) Cytokine gene polymorphism in human disease: on-line databases. Genes Immun 1: 3–19. 11197303 10.1038/sj.gene.6363645

[36] Ngoc LP, Gold DR, Tzianabos AO, Weiss ST, Celedón JC (2005) Cytokines, allergy, and asthma. Curr Opin Allergy Clin Immunol 161–166. 10.1097/01.all.0000162309.97480.45 15764907

[37] Wine E, Mack DR, Hyams J, Otley AR, Markowitz J, et al (2013) Interleukin-6 is associated with steroid resistance and reflects disease activity in severe pediatric ulcerative colitis. J Crohns Colitis S1873-9946: 00508–9. 10.1016/j.crohns.2012.12.012 23339932

[38] Postal M, Peliçari KO, Sinicato NA, Marini R, Costallat LT, et al.. (2013) Th1/Th2 cytokine profile in childhood-onset systemic lupus erythematosus. CytokineS1043-4666: 00806-X. 10.1016/j.cyto.2012.11.023 23332615

[39] Du Y, Dodel RC, Eastwood BJ, Bales KR, Gao F, et al (2000) Association of an interleukin 1-alpha polymorphism with Alzheimer's disease. Neurology 55: 480–484. 10953177 10.1212/wnl.55.4.480

[40] Grimaldi LM, Casadei VM, Ferri C, Veglia F, Licastro F, et al (2000) Association of early-onset Alzheimer's disease with an interleukin-1-alpha gene polymorphism. Ann Neurol 47: 361–365. 10716256

[41] Nicoll JAR, Mrak RE, Graham DI, Stewart J, Wilcock G, et al (2000) Association of interleukin-1 gene polymorphisms with Alzheimer's disease. Ann Neurol 47: 365–368. 10716257 PMC3833599

[42] Goedecke V, Crane AM, Jaakkola E, Kaluza W, Laiho K, et al (2003) Interleukin 10 polymorphisms in ankylosing spondylitis. Genes Immun 4: 74–76. 12595905 10.1038/sj.gene.6363930

[43] Hunninghake GM, Soto-Quirós ME, Lasky-Su J, Avila L, Ly NP, et al (2008) Dust mite exposure modifies the effect of functional IL10 polymorphisms on allergy and asthma exacerbations. J Allergy Clin Immunol 122: 93–98. 18440625 10.1016/j.jaci.2008.03.015 PMC6124308

[44] Shaker OG, Sadik NAH, El-Hamid NA (2013) Impact of single nucleotide polymorphism in tumor necrosis factor-α gene 308 G/A in Egyptian asthmatic children and wheezing infants. Hum Immunol S0198-8859: 00015–3. 10.1016/j.humimm.2013.01.004 23376082

[45] Thompson ME, Barkhuizen A (2003) Fibromyalgia, hepatitis C infection, and the cytokine connection. Curr Pain Headache Rep 7: 342–347. 12946286 10.1007/s11916-003-0032-2

[46] Balakrishnan VS, Guo D, Rao M, Jaber BL, Tighiouart H, et al (2004) Cytokine gene polymorphisms in hemodialysis patients: association with comorbidity, functionality, and serum albumin. Kidney Int 65: 1449–1460. 15086488 10.1111/j.1523-1755.2004.00531.x

[47] Rao M, Wong C, Kanetsky P, Girndt M, Stenvinkel P, et al (2007) Cytokine gene polymorphism and progression of renal and cardiovascular diseases. Kidney Int 72: 549–556. 17579660 10.1038/sj.ki.5002391

[48] Lord PCW, Wilmoth LM, Mizel SB, McCall CE (1991) Expression of interleukin-1 alpha and beta genes by human blood polymorphonuclear leukocytes. J Clin Invest 87: 1312–1321. 2010544 10.1172/JCI115134 PMC295162

[49] Karjalainen J, Hulkkonen J, Pessi T, Huhtala H, Nieminen MM, et al (2002) The IL1A genotype associates with atopy in nonasthmatic adults. J Allergy Clin Immunol 110: 429–434. 12209090 10.1067/mai.2002.126784

[50] Jongh CM, John SM, Bruynzeel DP, Calkoen F, Van Dijk FJ, et al (2008) Cytokine gene polymorphisms and susceptibility to chronic irritant contact dermatitis. Contact Dermatitis 58: 269–77. 18416756 10.1111/j.1600-0536.2008.01317.x

[51] Lowenthal JW, Zubler RH, Nabholz M, MacDonald HR (1985) Similarities between interleukin-2 receptor number and affinity on activated B and T lymphocytes. Nature 315: 669–672. 3925347 10.1038/315669a0

[52] Smith KA, Cantrell DA (1985) Interleukin 2 regulates its own receptors. Proc Natl Acad Sci USA 82: 864–868. 2983318 10.1073/pnas.82.3.864 PMC397147

[53] Christensen U, Haagerup A, Binderup HG, Vestbo J, Kruse TA, et al (2006) Family based association analysis of the IL2 and IL15 genes in allergic disorders. Eur J Hum Genet 14: 227–235. 16333313 10.1038/sj.ejhg.5201541

[54] Franceschi DAS, Viel DO, Sell AM, Tsuneto LT, Visentainer JEL (2009) Optimization of the PCR-SSP methodology in the identification of TNF and IL2 genetic polymorphisms. Rev Bras Hematol Hemoter 31: 241–246.

[55] Hoffmann SC, Stanley EM, Darrin Cox E, Craighead N, DiMercurio BS, et al (2001) Association of cytokine polymorphic inheritance and in vitro cytokine production in anti-CD3/CD28-stimulated peripheral blood lymphocytes. Transplantation 72: 1444–1450. 11685118 10.1097/00007890-200110270-00019

[56] Lu MP, Chen RX, Wang ML, Zhu XJ, Zhu LP, et al (2011) Association study on IL4, IL13 and IL4RA polymorphisms in mite-sensitized persistent allergic rhinitis in a Chinese population. PloS One 6: e27363. 22087298 10.1371/journal.pone.0027363 PMC3210163

[57] Micheal S, Minhas K, Ishaque M, Ahmed F, Ahmed A (2013) IL-4 gene polymorphisms and their association with atopic asthma and allergic rhinitis in Pakistani patients. J Investig Allergol Clin Immunol 23: 107–111. 23654077

[58] Vishnumaya CP, Sudha S, Suhail N, Gemitha G, Saranya RS, et al (2013) Association of -590C/T interleukin-4 gene promoter polymorphism with atopic asthma in south indian people. Int. J. Bioassays 2: 723–726.

[59] Hosseini-Farahabadi S, Tavakkol-Afshari J, Rafatpanah H, Farid Hosseini R, Khaje Daluei M (2007) Association between the polymorphisms of IL-4 gene promoter (-590C>T), IL-13 coding region (R130Q) and IL-16 gene promoter (-295T>C) and allergic asthma. Iran J Allergy Asthma Immunol 6: 9–14. 17303923

[60] Liu X, Beaty TH, Deindl P, Huang SK, Lau S, et al (2004) Associations between specific serum IgE response and 6 variants within the genes *IL4*, *IL13*, and *IL4RA* in German children: The German Multicenter Atopy Study. J Allergy Clin Immunol 113: 489–495. 15007352 10.1016/j.jaci.2003.12.037

[61] Landeck L, Visser M, Kezic S, John SM (2013) IL1A-889 C/T gene polymorphism in irritant contact dermatitis. J Eur Acad Dermatol Venereol 27: 1040–1043. 22364598 10.1111/j.1468-3083.2012.04474.x

[62] Padrón-Morales J, Sanz C, Dávila I, Muñoz-Bellido F, Lorente F, et al (2013) Polymorphisms of the IL12B, IL1B, and TNFA Genes and Susceptibility to Asthma. J Investig Allergol Clin Immunol 23: 487–494. 24654313

[63] Atanasovska-Stojanovska A, Popovska M, Trajkov D, Spiroski M (2013) IL1 cluster gene polymorphisms in Macedonian patients with chronic periodontitis. Bratisl Lek Listy 114: 380–385. 23822621 10.4149/bll_2013_080

[64] Amirzargar AA, Rezaei N, Jabbari H, Danesh AA, Khosravi F, et al (2006) Cytokine single nucleotide polymorphisms in Iranian patients with pulmonary tuberculosis. Eur Cytokine Netw 17: 84–89. 16840026

[65] Tahmasebi Z, Akbarian M, Mirkazemi S, Shahlaee A, Alizadeh Z, et al (2013) Interleukin-1 gene cluster and IL-1 receptor polymorphisms in Iranian patients with systemic lupus erythematosus. Rheumatol Int 33: 2591–2596. 23722873 10.1007/s00296-013-2784-2

[66] Miyake Y, Tanaka K, Arakawa M (2013) Case-control study of eczema in relation to IL4Rα genetic polymorphisms in Japanese women: The Kyushu Okinawa Maternal and Child Health Study. Scand J Immunol 77: 413–418. 23480403 10.1111/sji.12043

[67] Chen T, Liang W, Gao L, Wang Y, Liu Y, et al (2011) Association of single nucleotide polymorphisms in interleukin 12 (IL-12A and -B) with asthma in a Chinese population. Hum Immunol 72: 603–606. 21513752 10.1016/j.humimm.2011.03.018

[68] Chong WP, Ip WK, Tso GH, Ng MW, Wong WH, et al (2006) The interferon gamma gene polymorphism +874 A/T is associated with severe acute respiratory syndrome. BMC Infect Dis 6: 82. 16672072 10.1186/1471-2334-6-82 PMC1468415

[69] Celedón JC, Lange C, Raby BA, Litonjua AA, Palmer LJ, et al (2004) The transforming growth factor-beta1 (TGFB1) gene is associated with chronic obstructive pulmonary disease (COPD). Hum Mol Genet 15: 1649–1656. 10.1093/hmg/ddh171 15175276

[70] Sharma S, Raby BA, Hunninghake GM, Soto-Quirós M, Avila L, et al (2009) Variants in TGFB1, dust mite exposure, and disease severity in children with asthma. Am J Respir Crit Care Med 179: 356–362. 19096005 10.1164/rccm.200808-1268OC PMC2648908

[71] MfunaEndam L, Cormier C, Bossé Y, Filali-Mouhim A, Desrosiers M (2010) Association of IL1A, IL1B, and TNF gene polymorphisms with chronic rhinosinusitis with and without nasal polyposis: A replication study. Arch Otolaryngol Head Neck Surg 136: 187–192. 20157068 10.1001/archoto.2009.219

[72] Lin YJ, Wan L, Sheu JJ, Huang CM, Lin CW, et al (2008) G/T polymorphism in the interleukin-2 exon 1 region among Han Chinese systemic lupus erythematosus patients in Taiwan. Clin Immunol 129: 36–39. 18650128 10.1016/j.clim.2008.05.011

[73] Howson JM, Cooper JD, Smyth DJ, Walker NM, Stevens H, et al (2012) Evidence of gene-gene interaction and age-at-diagnosis effects in type 1 diabetes. Diabetes 61: 3012–3017. 22891215 10.2337/db11-1694 PMC3478521

[74] Hussain SK, Madeleine MM, Johnson LG, Du Q, Malkki M, et al (2008) Cervical and vulvar cancer risk in relation to the joint effects of cigarette smoking and genetic variation in interleukin 2. Cancer Epidemiol Biomarkers Prev. 17: 1790–1799. 10.1158/1055-9965.EPI-07-2753 PMC2497438 18628433

[75] Movahedi M, Amirzargar AA, Nasiri R, Hirbod-Mobarakeh A, Farhadi E, et al (2013) Gene polymorphisms of Interleukin-4 in allergic rhinitis and its association with clinical phenotypes. Am J Otolaryngol 34: 676–681. 24075353 10.1016/j.amjoto.2013.05.002

[76] Baye TM, Butsch Kovacic M, Biagini Myers JM, Martin LJ, Lindsey M, et al (2011) Differences in candidate gene association between European ancestry and African American asthmatic children. PLoSOne 6: e16522. 10.1371/journal.pone.0016522 PMC3046166 21387019

[77] Jeng JR, Wang JH, Liu WS, Chen SP, Chen MY, et al (2005) Association of interleukin-6 gene G-174C polymorphism and plasma plasminogen activator inhibitor-1 level in Chinese patients with and without hypertension. Am J Hypertens 18: 517–522. 15831362 10.1016/j.amjhyper.2004.10.028

[78] Illig T, Bongardt F, Schöpfer A, Müller-Scholze S, Rathmann W, et al (2004) Significant association of the interleukin-6 gene polymorphisms C-174G and A-598G with type 2 diabetes. J Clin Endocrinol Metab 89: 5053–5058. 15472205 10.1210/jc.2004-0355

[79] Fishman D, Faulds G, Jeffery R, Mohamed-Ali V, Yudkin JS, et al (1998) The effect of novel polymorphisms in the interleukin-6 (IL-6) gene on IL-6 transcription and plasma IL-6 levels, and an association with systemic-onset juvenile chronic arthritis. J Clin Invest 102: 1369–1376. 9769329 10.1172/JCI2629 PMC508984

[80] Maitra A, Shanker J, Dash D, John S, Sannappa PR, et al (2008) Polymorphisms in the IL6 gene in Asian Indian families with premature coronary artery disease–the Indian Atherosclerosis Research Study. Thromb Haemost 99: 944–950. 18449426 10.1160/TH07-11-0686

[81] Campos Alberto EJ, Shimojo N, Suzuki Y, Mashimo Y, Arima T, et al (2008) IL-10 gene polymorphism, but not TGF-beta1 gene polymorphisms, is associated with food allergy in a Japanese population. Pediatr Allergy Immunol 19: 716–721. 18208460 10.1111/j.1399-3038.2007.00709.x

[82] Chelluri EP, Chelluri LK, Pawar S, Debnath T, Reddy SR (2013) IL-10 polymorphisms in inflammatory and infectious respiratory diseases with seropositive H. Pylori. IJRRMS 3: 5–10.

[83] Jacob CM, Pastorino AC, Okay TS, Castro AP, Gushken AK, et al (2013) Interleukin 10 (IL10) and transforming growth factor β1 (TGFβ1) gene polymorphisms in persistent IgE-mediated cow's milk allergy. Clinics 68: 1004–9. 23917667 10.6061/clinics/2013(07)19 PMC3714916

